# Post-thaw quality of ram sperm frozen with different concentrations of low-density lipoproteins associated with non-enzymatic antioxidants

**DOI:** 10.1590/1984-3143-AR2022-0068

**Published:** 2023-04-21

**Authors:** Paola Pereira das Neves Snoeck, Diogo Ribeiro Câmara, Luís Cláudio de Oliveira Moura, Maíra Corona da Silva, Mariana Machado-Neves, Milton Rezende Teixeira-Neto, Marc Henry

**Affiliations:** 1 Departamento de Ciências Agrárias e Ambientais, Universidade Estadual de Santa Cruz, Ilhéus, BA, Brasil; 2 Departamento de Medicina Veterinária, Universidade Federal de Alagoas, Viçosa, AL, Brasil; 3 Departamento de Biologia Geral, Universidade Federal de Viçosa, Viçosa, MG, Brasil; 4 Departamento de Medicina Veterinária, Centro Universitário UNIFTC, Vitória da Conquista, BA, Brasil; 5 Departamento de Clínica e Cirurgia Veterinárias, Universidade Federal de Minas Gerais, Belo Horizonte, MG, Brasil

**Keywords:** ascorbyl palmitate, cryopreservation, hydroxytoluene butylate, sheep, spermatozoa

## Abstract

The cryopreservation reduces ram sperm quality, decreasing the pregnancy rate of ewes inseminated with thawed sperm. Hence, we aimed to improve the post-thaw quality of ram sperm replacing egg yolk on Tris-Glucose extender with different concentrations of LDL (2 or 8%), associated with the addition of 10 mM non-enzymatic antioxidants (ascorbic acid, hydroxytoluene butylate, ascorbyl palmitate, and trehalose). Semen samples were collected from six rams, split into different treatments, and frozen. After thawing, kinematic (CASA), structural (propidium iodide and carboxyfluorescein diacetate) and functional (hypoosmotic test) sperm membrane integrity was assessed. Total motility, VCL, and LIN were also assessed in thawed samples during 3 h of incubation (38 °C). The results showed that hydroxytoluene butylate at 10 mM in Tris-Glucose extender with 8% LDL improved velocity parameters immediately post-thaw compared with Tris-Glucose egg yolk extender, as well as prevented the reduction of total motility and VCL after incubation. There was no benefit of adding ascorbic acid and trehalose. Moreover, for the first time, it was shown the motility impairment promoted by ascorbyl palmitate to ram sperm.

## Introduction

The effectiveness of transcervical artificial insemination (AI) in sheep using frozen-thawed semen is still low (Marti et al., 2008; Tonieto et al., 2010). The damages observed in frozen-thawed ram sperm involve changes in cell structure and function that negatively impact fertility (Peris-Frau et al., 2020). Among cryoinjuries, it is possible to highlight the lipid transition phase of the cell membrane at low temperatures (Öztürk et al., 2020), the imbalance between the production of reactive oxygen species (ROS) and efficiency of the antioxidant defense system (Ball, 2008), and the osmotic stress (Morris et al., 2007; Sieme et al., 2015).

Different components have been added to semen extenders to improve the quality of frozen-thawed spermatozoa (Sieme et al., 2015; Peris-Frau et al., 2020). Lyophilized egg yolk, plasma egg yolk, and low-density lipoprotein (LDL) have been successfully used for ram semen cryopreservation (Tonieto et al., 2010; Silva et al., 2014; Alcay et al., 2015), and the egg yolk replacement by LDL has shown similar or superior cryoprotective effects in frozen-thawed spermatozoa from several species (Hu et al., 2010, 2011; Moustacas et al., 2011; Vera-Munoz et al., 2011; Neves et al., 2014; Varela et al., 2020). Overall, LDL favors sperm membrane stabilization, protecting the membrane from lipid peroxidation, and increasing the activity of enzymatic antioxidants in thawed sperm (Brouwers and Gadella, 2003; Bergeron et al., 2005; Hu et al., 2011; Pini et al., 2018; Belala et al., 2019).

Furthermore, sperm membranes are enriched with polyunsaturated fatty acids (Tremellen, 2008; Peris-Frau et al., 2020) that are easily oxidized by free radicals and peroxides, reducing the motility and activating the apoptotic process, which might culminate in DNA fragmentation (Bast et al., 1991; Baldi et al., 2020). Although ROS generation is primarily required for physiological signaling pathways, a precise balance between its production and scavenging is crucial for sperm viability (Bast et al., 1991; Gibb et al., 2020). Therefore, the addition of antioxidants to the extender during semen cryopreservation has been tested in several species to improve semen quality, including in sheep (Cirit et al., 2013; Snoeck et al., 2015; Amidi et al., 2016; Fang and Zhong, 2020; Galarza et al., 2020).

In this framework, this study aimed at evaluating the protective effect of non-enzymatic antioxidants added into LDL extenders on sperm quality after the freezing-thawing process. For this purpose, two extenders were selected and used in two Experiments (I and II), to evaluate the influence of adding non-enzymatic antioxidants (ascorbic acid, ascorbyl palmitate, hydroxytoluene butylate, and trehalose) on the post-thaw sperm quality.

## Methods

### Ethics statement

All the experimental protocols and animal care were approved by the Commission of Ethics in Animal Experimental of the State University of Santa Cruz (number 007/10).

### Animals, semen collection and evaluation

Six Santa Inês rams (2-5 years of age), a Brazilian hair breed with a low degree of reproductive seasonality (Balaro et al., 2014), were used. The animals were at College of Veterinary Medicine, Federal University of Minas Gerais, Brazil (19° 86′ 94″ south, 43° 96′ 84″ west) with uniform feeding, housing, and lighting conditions, under regular semen collection (three times/week) using artificial vagina during the whole experimental period. Minimal requirements to freeze ejaculates were: volume ≥ 0.5 mL, motility ≥ 75%, mass motility ≥ 3, morphologically normal sperm ≥ 70%, and a minimum of ≥ 1,8 x 10^9^ sperm/ejaculate.

### Extenders

Tris-Glucose extender was used as the base extender medium and was composed of 3.69 g Tris, 1.99 g citric acid, 0.5 g glucose, 100 mg streptomycin, 100,000 UI penicillin, and bi-distilled water (qsp 100 mL). When 16% egg yolk (v:v) and 5% glycerol (v:v) were added, it was named Control (C; 1,241 mOsmol/L). In turn, the LDL used to replace egg yolk in the modified Tris-Glucose was extracted using the method described by Neves et al. (2014).

Further, a previous experiment was conducted to determine the best concentrations of LDL and glycerol to be used in the modified Tris-Glucose extender. For that, ram sperm were frozen in extenders containing LDL at 2, 8, and 16%, and glycerol at 3, 5, and 7%. The highest total motility immediately post-thaw was observed in spermatozoa preserved within the extender containing 5% glycerol and 2% LDL (1,208 mOsmol/L; G5L2), whereas the best motility stability was obtained after 3 h of incubation in frozen-thawed sperm using the extender containing 5% glycerol and 8% LDL (1,165 mOsmol/L; G5L8). In addition, those extenders were capable to preserve the structural and functional integrity of sperm membranes (see results in Supplementary Material). Therefore, the extenders G5L2 and G5L8 were selected to be used in the Experiments I and II, respectively.

In those experiments, each ejaculate from individual rams was aliquoted into G5L2 and G5L8 extenders added with 10 mM of four non-enzymatic antioxidants: ascorbic acid (AA), hydroxytoluene butylate (HB), ascorbyl palmitate (AP), and trehalose (TH). In summary, the quality of post-thaw ram sperm was assessed using the following extenders (Experiment I): Control (C); G5L2; G5L2 + AA; G5L2 + HB; G5L2 + AP; and G5L2 + TH. In the Experiment II, the following extenders used were: C; G5L8; G5L8 + AA; G5L8 + HB; G5L8 + AP; and G5L8 + TH.

### Semen cryopreservation and thawing

Semen samples were diluted in their respective treatments for each experiment with a final concentration of 100 x 10^6^ motile sperm/mL. Extended semen samples were packed into 0.25 mL straws (IMV Technology, L’Aigle, Cedex, France), cooled to 5 °C at a rate of −0.25 °C/min, maintained at 5 °C for 2 h, thereafter a freezing rate of −25 °C/min was used from 5 to -140 °C using the equipment TK4000^®^ (TK Tecnologia, Uberaba, MG, Brazil). Upon reaching −140 °C the straws were transferred to liquid nitrogen (−196 °C) and stored until evaluation.

### Post-thaw sperm evaluation

All samples were thawed at 38 °C for 30 s and kept in a water bath for 5 min before analysis. Spermatozoa were evaluated for kinematics (Computer Assisted Sperm Analysis, CASA, SCA^®^, v.4, Microptics S.L., Barcelona, Spain), functional integrity of plasma membrane (Hypoosmotic Swelling Test, HOST), and structural integrity of plasma and acrosomal membranes (propidium iodide and carboxyfluorescein diacetate, PI/CFDA).

Before CASA analysis, thawed samples were diluted in C media (38 °C) to obtain a final concentration of 50 x 10^6^ sperm/mL. The following endpoints were determined: total motility (TM, %), progressive motility (PM, %), curvilinear velocity (VCL, μm/s), progressive velocity (VSL, μm/s), path velocity (VAP, μm/s), linearity (LIN, %), and amplitude of lateral head displacement (ALH, μm/s). Kinematic endpoints were measured with the following settings: particle area 9-70 μm; progressivity >80% of STR; circular < 50% LIN; 25 images/s. At least three randomly selected microscopic fields were scanned. A minimum of 500 sperm/treatment were recorded.

For the HOST analysis, aliquots (25 µL) of each sample were diluted in 200 µL of fructose- citrate solution (100 mOsmol/L). After 30 min of incubation (38 °C) the samples were fixed with formalin-buffered saline (300 µL) and 100 sperm were evaluated (× 1000 magnification; Olympus^®^ BX 41). The percentage of reactive cells was calculated according to Melo et al. (2005). Structural integrity of plasma and acrosomal membranes was determined according to Harrison and Vickers (1990). Under an epifluorescence microscope (× 400 magnification; Olympus^®^ CX 51) and 200 sperm/treatment were evaluated. Cells were classified into three subpopulations: intact (plasma and acrosomal membranes integrity, PI^-^CFDA^+^), partially intact (plasma membrane damaged and intact acrosome, PI^+^CFDA^+^), damaged (both plasma and acrosomal membranes damaged, PI^+^CFDA^-^). To evaluate the effectiveness of the treatments only the percentage of intact sperm was considered.

Finally, after thawing, aliquots of all the samples from the Experiments I and II were maintained in a dry bath (38 °C), and total motility, VCL, and LIN were assessed by CASA after 1, 2, and 3 h of incubation, to determine sperm longevity during incubation.

### Statistical analysis

The experimental designed was completely randomized block, in a 6 × 6 factorial test (6 rams × 6 treatments), eliminating the ram influence between treatments (St-Pierre, 2007). For all Experiments, three ejaculates (replicates) from each ram were collected, aliquoted into different extenders tested, and frozen individually. The results were grouped, totaling 18 samples/treatment/Experiment. Analysis of variance (ANOVA) was performed, and the means were compared using Duncan’s test. Data homoscedasticity (Levene’s test) and normality were assessed (Kolmogorov-Smirnov and Shapiro-Wilk tests). Data not normally distributed were arcsine transformed before analysis. As each Experiment was performed separately, no comparisons between Experiments were made, since different periods of semen collection can affect semen quality and freezability (Martínez-Fresneda et al., 2019; Freitas et al., 2020; Landaeta-Hernández et al., 2020;). Data are presented non-transformed (mean ± SEM). A probability of P < 0.05 was considered significant.

## Results

### Experiment I

The extender containing G5L2+AP presented the lowest TM immediately post-thaw (P < 0.05), but no difference was observed among other treatments. The PM of post-thaw spermatozoa was lower in the extenders G5L2+AP and G5L2+TH when compared to G5L2 and G5L2+ HB (P < 0.05), which presented the highest PM, followed by the G5L2+AA and control extenders. For the remaining kinematic parameters, the extender G5L2+AP showed the lowest values (P < 0.05). By contrast, the extender G5L2+HB presented the highest means for VSL, VAP, and LIN (Table 1).

**Table 1:** Post-thaw kinematic parameters of ram sperm (mean ± SEM) frozen in Tris-glucose base extender containing 16% egg yolk and 5% glycerol (Control) or using base extender with no egg yolk, 5% glycerol and 2% low-density lipoprotein (G5L2) with addition of different non-enzymatic antioxidants (10 mM).

**Extenders**	**Parameters**						
	**TM (%)**	**PM (%)**	**VCL (µm/s)**	**VSL (µm/s)**	**VAP (µm/s)**	**LIN (%)**	**ALH (µm)**
Control	36.1±3.7^a^	9.8±1.6^ab^	39.7±1.5^a^	16.5±1.0^b^	22.6±1.1^b^	41.0±1.3^ab^	2.9±0.1^a^
G5L2	34.9±4.3^a^	11.2 ± 2.1^a^	43.3±1.9^a^	18.8±1.4^ab^	25.8±1.7^ab^	42.6±1.6^ab^	2.8±0.1^a^
G5L2+AA	34.1±2.9^a^	10.1±1.4^ab^	42.3±1.7^a^	17.2±1.2^ab^	23.3±1.3^ab^	39.9±1.6^b^	3.1±0.1^a^
G5L2+HB	35.8±3.3^a^	11.8±1.7^a^	43.8±2.1^a^	20.1±1.5^a^	27.2±1.8^a^	45.3±2.2^a^	2.8±0.1^a^
G5L2+AP	5.7±0.6^b^	0.0±0.0^c^	15.4±0.7^b^	2.9±0.3^c^	5.9±0.5^c^	18.2±1.9^c^	0.2±0.1^b^
G5L2+TH	28.0±2.5^a^	6.9±1.0^bc^	38.7±1.8^a^	15.4±1.3^b^	21.7±1.6^b^	38.8±2.0^b^	3.1±0.1^a^

AA: ascorbic acid; HB: hydroxytoluene butylate; AP: ascorbyl palmitate; TH: trehalose; TM: total motility; PM: progressive motility; VCL: curvilinear velocity; VSL: progressive velocity; VAP: path velocity; LIN: linearity; ALH: amplitude of lateral head displacement. Within columns, means with no common superscript letters are different (P < 0.05).

Throughout the incubation time at 38 °C, TM, VCL, and LIN of sperm diluted in the extender G5L2+AP were lower than other treatments and no longer detected at 2 and 3 h. The addition of non-enzymatic antioxidants AA, HB, and TH did not prevent the reduction in the percentage of TM during 3 h of incubation, as only ram sperm diluted in the extender G5L2 showed no significant reduction in their TM during incubation. Moreover, the extender G5L2 was better than G5L2+AP or G5L2+TH after 3 h of incubation (P < 0.05; Table 2).

**Table 2:** Kinematic parameters of ram sperm (mean ± SEM) frozen in Tris-glucose base extender containing 16% egg yolk and 5% glycerol (Control) or using base extender with no egg yolk, 5% glycerol and 2% low-density lipoprotein (G5L2) with addition of different non-enzymatic antioxidants (10 mM) during 3h of incubation at 38 °C after thawing.

	**Extender**	**Incubation time**			
		**0h**	**1h**	**2h**	**3h**
**TM (%)**	Control	36.1 ± 3.7^a^	34.3 ± 3.8^ab^	27.2 ± 3.1^a^	20.4 ± 3.0^ab^*
	G5L2	34.9 ± 4.3^a^	33.4 ± 3.3^ab^	27.1 ± 3.0^a^	25.1 ± 3.1^a^
	G5L2 + AA	34.1 ± 2.9^a^	30.4 ± 2.6^ab^	23.6 ± 2.8^a*^	19.8 ± 2.3^ab*^
	G5L2 + HB	35.8 ± 3.3^a^	36.5 ± 3.8^a^	25.4 ± 2.1^a*^	22.7 ± 2.5^ab*^
	G5L2 + AP	5.7 ± 0.6^c^	5.2 ± 0.9^c^	0.0 ± 0.0^c*^	0.0 ± 0.0^c*^
	G5L2 + TH	28.0 ± 2.5^ab^	26.5 ± 2.5^b^	21.3 ± 2.1^a*^	17.1 ± 1.4^b*^
**VCL (µm/s)**	Control	39.7 ± 1.5^a^	39.9 ± 1.9^a^	33.8 ± 2.3^a*^	26.7 ± 2.0^b*^
	G5L2	43.3 ± 1.9^a^	41.1 ± 2.2^a^	38.0 ± 2.4^a*^	32.4 ± 2.2^a*^
	G5L2 + AA	42.3 ± 1.7^a^	40.4 ± 2.3^a^	33.4 ± 2.0^a*^	26.4 ± 1.7^b*^
	G5L2 + HB	43.8 ± 2.1^a^	44.5 ± 1.8^a^	39.5 ± 1.8^a^	33.2 ± 2.2^a*^
	G5L2 + AP	15.4 ± 0.7^b^	14.9 ± 2.0^b^	0.0 ± 0.0^b*^	0.0 ± 0.0^c*^
	G5L2 + TH	38.7 ± 1.8^a^	38.4 ± 2.1^a^	34.0 ± 2.3^a^	30.1 ± 1.6^ab*^
**LIN (%)**	Control	41.0 ± 1.3^ab^	36.9 ± 1.2^a^	35.8 ± 1.3^a^	31.1 ± 1.7^a*^
	G5L2	42.6 ± 1.6^ab^	34.2 ± 1.2^a^	31.2 ± 2.0^ab^	29.1 ± 2.3^ab*^
	G5L2 + AA	39.9 ± 1.6^b^	34.5 ± 1.7^a^	30.0 ± 1.5^b^	25.5 ± 2.5^bc*^
	G5L2 + HB	45.3 ± 2.2^a^	35.8 ± 1.3^a^	34.7 ± 1.6^a^	31.2 ± 2.0^a*^
	G5L2 + AP	18.2 ± 1.9^c^	12.8 ± 1.7^c^	0.0 ± 0.0^c^	0.0 ± 0.0^d*^
	G5L2 + TH	38.8 ± 2.0^b^	29.3 ± 1.4^b^	27.9 ± 1.8^b^	23.6 ± 1.9^c*^

Within columns, means with no common superscript letters are different (P < 0.05). *Indicates reduction within treatment during incubation time (P < 0.05).

**Figure 1:**
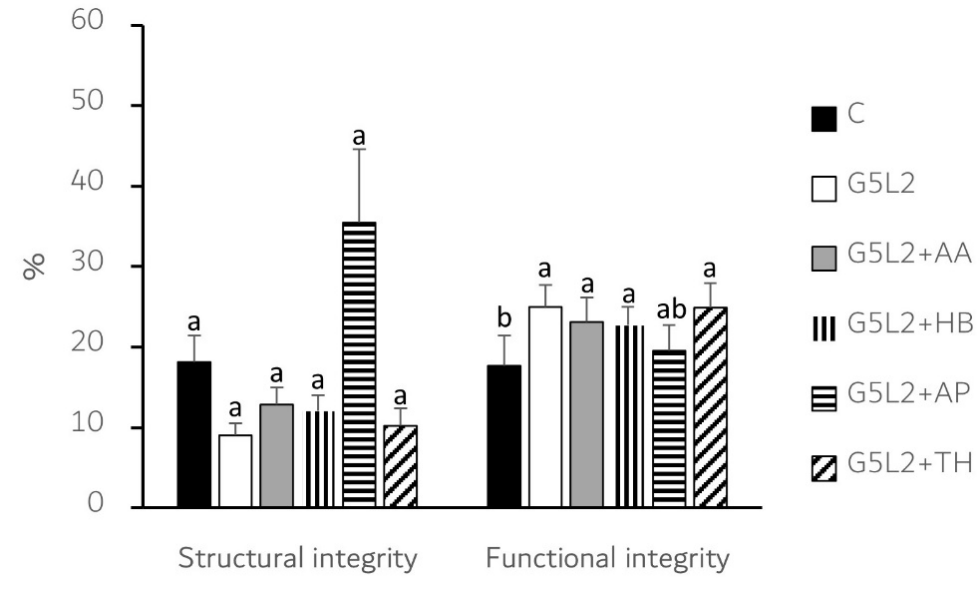
Experiment I ram sperm frozen in Tris-glucose base extender containing 16% egg yolk and 5% glycerol (Control, C) or using base extender with no egg yolk, 5% glycerol and 2% low-density lipoprotein (G5L2) with addition of different non-enzymatic antioxidants (10 mM). Structural (IP^-^CFDA^+^) and functional integrity (hypoosmotic test) post-thaw. AA: ascorbic acid; HB: hydroxytoluene butylate; AP: ascorbyl palmitate; TH: trehalose. Different letters indicate differences among treatments (P < 0.05).

Finally, the structural integrity of sperm membranes was not enhanced by the addition of non-enzymatic antioxidants in the extender G5L2 (P > 0.05). Conversely, the functional integrity of ram sperm was lower in the C than most of the extenders tested, except for G5L2+AP (P < 0.05; Figure 1).

### Experiment II

The extenders G5L8 and G5L8+HB elicited higher TM and PM post-thaw, being superior to C in most of kinematic parameters, except for LIN and ALH (P <0,05). Conversely, the ram sperm cryopreserved in the extender G5L8+AP presented the lowest means for all kinematic endpoints (Table 3).

**Table 3:** Post-thaw kinematic parameters of ram sperm (mean ± SEM) frozen in Tris-glucose base extender containing 16% egg yolk and 5% glycerol (Control) or using base extender with no egg yolk, 5% glycerol and 8% low-density lipoprotein (G5L8) with addition of different non-enzymatic antioxidants (10 mM).

**Extenders**	**Parameters**						
	**TM (%)**	**PM (%)**	**VCL (µm/s)**	**VSL (µm/s)**	**VAP (µm/s)**	**LIN (%)**	**ALH (µm)**
Control	31.9±3.4^c^	9.4±1.9^b^	36.6±2.3^b^	16.0±1.6^c^	21.3±1.8^c^	42.3±2.0^b^	2.7±0.1^a^
G5L8	49.5±3.2^a^	18.3±1.8^a^	48.8±2.0^a^	22.6±1.3^ab^	30.2±1.5^ab^	45.7±1.3^ab^	2.8±0.0^a^
G5L8 + AA	37.7±3.4^bc^	13.4±1.9^ab^	47.5±2.0^a^	20.8±1.4^ab^	27.7±1.6^b^	43.3±1.6^b^	2.9±0.1^a^
G5L8 + HB	46.3±4.6^ab^	19.7±3.3^a^	50.5±2.4^a^	25.1±1.7^a^	33.3±2.1^a^	49.0±1.7^a^	2.7±0.1^a^
G5L8 + AP	15.2±0.4^d^	0.3±0.1^c^	19.8±0.8^c^	4.7±0.4^d^	9.0±0.6^d^	23.6±1.3^c^	0.5±0.2^b^
G5L8 + TH	36.3±4.5^c^	11.5±1.8^b^	43.7±2.1^a^	18.6±1.5^bc^	25.4±1.7^bc^	41.6±1.7^b^	3.1±0.1^a^

AA: ascorbic acid; HB: hydroxytoluene butylate; AP: ascorbyl palmitate; TH: trehalose; TM: total motility; PM: progressive motility; VCL: curvilinear velocity; VSL: progressive velocity; VAP: path velocity; LIN: linearity; ALH: amplitude of lateral head displacement. Within columns, means with no common superscript letters are different (P < 0.05).

After 3 h of incubation, the TM of spermatozoa cryopreserved in the extenders containing G5L8 and G5L8+HB were equivalent, being superior to extenders C, G5L8+AP, and G5L8+TH (P < 0.05). It is noteworthy that extenders C and G5L8+HB did not exhibit a significant reduction in TM during the incubation period. The addition of AP in the extender with G5L8 reduced the TM, VCL, and LIN to zero after 2 h of incubation (Table 4).

**Table 4:** Kinematic parameters of ram sperm (mean ± SEM) frozen in Tris-glucose base extender containing 16% egg yolk and 5% glycerol (Control) or using base extender with no egg yolk, 5% glycerol and 8% low-density lipoprotein (G5L8) with addition of different non-enzymatic antioxidants (10 mM) during 3h of incubation at 38 °C after thawing.

	**Extender**	**Incubation time**			
		**0h**	**1h**	**2h**	**3h**
**TM (%)**	Control	31.9 ± 3.4^c^	31.1 ± 3.6^b^	24.9 ± 3.8^c^	22.9 ± 3.4^c^
	G5L8	49.5 ± 3.2^a^	41.3 ± 3.7^ab^	39.9 ± 3.7^a^	34.3 ± 3.7^ab^*
	G5L8 + AA	37.7 ± 3.4^bc^	36.1 ± 2.9^ab^	30.4 ± 2.6^bc^	28.0 ± 2.3^bc*^
	G5L8 + HB	46.3 ± 4.6^ab^	43.5 ± 5.0^a^	38.5 ± 4.1^ab^	37.3 ± 4.3^a^
	G5L8 + AP	15.2 ± 0.4^d^	9.2 ± 1.0^c^	0.0 ± 0.0^d^	0.0 ± 0.0^d*^
	G5L8 + TH	36.3 ± 4.5^c^	33.3 ± 3.7^ab^	25.6 ± 2.6^c^	20.6 ± 2.1^c*^
**VCL (µm/s)**	Control	36.6 ± 2.3^b^	41.3 ± 2.5^c^	32.3 ± 2.2^d^	28.8 ± 2.5^b*^
	G5L8	48.8 ± 2.0^a^	52.9 ± 2.5^ab^	50.1 ± 2.2^a^	45.5 ± 2.5^a*^
	G5L8 + AA	47.5 ± 2.0^a^	47.8 ± 2.2^ab^	45.0 ± 2.1^ab^	41.0 ± 2.5^a*^
	G5L8 + HB	50.5 ± 2.4^a^	54.4 ± 2.6^a^	47.6 ± 2.5^a^	47.0 ± 3.1^a^
	G5L8 + AP	19.8 ± 0.8^c^	16.6 ± 0.5^d^	0.0 ± 0.0^e^	0.0 ± 0.0^c*^
	G5L8 + TH	43.7 ± 2.1^a^	45.8 ± 2.5^bc^	40.2 ± 2.8^bc^	33.0 ± 2.2^b*^
**LIN (%)**	Control	42.3 ± 2.0^b^	36.4 ± 1.3^a^	33.2 ± 1.9^abc^	31.1 ± 2.5^b*^
	G5L8	45.7 ± 1.3^ab^	35.2 ± 1.2^ab^	36.2 ± 1.1^ab^	33.9 ± 0.9^ab*^
	G5L8 + AA	43.3 ± 1.6^b^	37.4 ± 1.3^a^	34.4 ± 1.3^abc^	35.7 ± 2.1^ab*^
	G5L8 + HB	49.0 ± 1.7^a^	37.8 ± 1.7^a^	37.5 ± 2.0^a^	37.1 ± 2.3^a*^
	G5L8 + AP	23.6 ± 1.3^d^	20.0 ± 1.2^c^	0.0 ± 0.0^d^	0.0 ± 0.0^c*^
	G5L8 + TH	41.6 ± 1.7^bc^	34.4 ± 1.3^ab^	32.4 ± 1.8^bc^	31.3 ± 2.3^b*^

Within columns, means with no common superscript letters are different (P < 0.05). *Indicates reduction within treatment during incubation time (P < 0.05).

**Figure 2:**
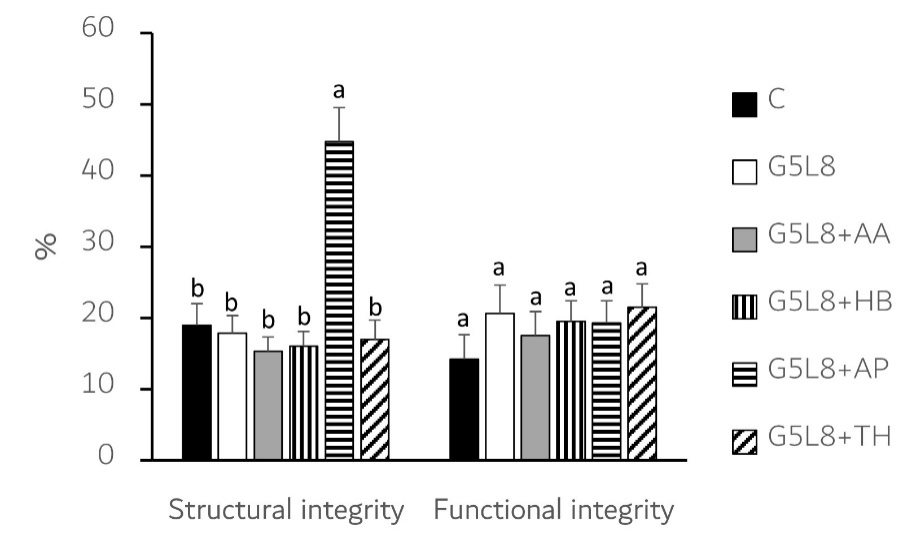
Experiment II ram sperm frozen in Tris-glucose base extender containing 16% egg yolk and 5% glycerol (Control, C) or using base extender with no egg yolk, 5% glycerol and 8% low-density lipoprotein (G5L8) with addition of different non-enzymatic antioxidants (10 mM). Structural (IP^-^CFDA^+^) and functional integrity (hypoosmotic test) post-thaw. AA: ascorbic acid; HB: hydroxytoluene butylate; AP: ascorbyl palmitate; TH: trehalose. Different letters indicate statistical differences between treatments (P < 0.05).

In contrast to the other treatments, the structural integrity of sperm membranes was better preserved when the extender containing G5L8+AP was used (P < 0.05). On the other hand, there were no differences among treatments on the preservation of sperm membrane functional integrity after the freezing-thawing process (Figure 2).

## Discussion

The efficacy of both 2 and 8% LDL instead of 16% egg yolk in Tris-Glucose base extender to cryopreserve ram sperm was demonstrated in the present study, corroborating previous reports (Tonieto et al., 2010; Moustacas et al., 2011; Vera-Munoz et al., 2011; Silva et al., 2014; Loaiza-Echeverri et al., 2015). It was shown that even at a low LDL concentration (2%) it was possible to obtain a high percentage of sperm with functional plasma membrane and post-thaw kinematic parameters similar or superior to Control extender. This fact probably reflects the effects of LDL as a cellular cryoprotectant (Belala et al., 2019). An interaction between LDL and “binder of sperm proteins (BSPs)” of ram seminal plasma was demonstrated, modulating cholesterol efflux, and promoting membrane stabilization (Bergeron et al., 2005; Sieme et al., 2015). This interaction remains active after the freezing-thawing process (Bergeron and Manjunath, 2006) and it was shown that BSPs improved ram sperm quality post-thaw (Pini et al., 2018), supporting the present results.

*In vitro* manipulations, such as temperature changes, removal of seminal plasma, and the freezing-thawing process are related to higher ROS production or reduction in semen antioxidant activity (Marti et al., 2008; Baldi et al., 2020). The effect of non-enzymatic antioxidants on extenders with LDL replacing egg yolk was evaluated in Experiments I and II. We recognize limitations in these Experiments that must be addressed. If ROS production, total antioxidant capacity, and sperm lipoperoxidation were measured in the different treatments, a better understanding of the influence on non-enzymatic antioxidants tested on sperm oxidative status could be assessed. Therein, to validate our results, we cannot overlook the importance of future studies with a larger number of sperm analysis using different antioxidant concentrations.

With regards to the non-enzymatic antioxidants added to extenders, it was noticed that HB was able to improve some post-thaw parameters. For Experiment I, it was observed that HB addition resulted in parameters similar to G5L2, but with higher VSL, VAP, and percentage of functional sperm membrane compared with the Control. For Experiment II, HB addition also presented post-thaw sperm parameters similar to G5L8, but it was superior to the Control in several endpoints immediately post-thaw (TM, PM, VSL, VAP, and LIN). Furthermore, unlike what was observed for the G5L8 extender, no significant reduction in total motility and VCL were observed after 3 h of incubation when HB was added. The better stability observed during incubation after adding HB is an important *in vitro* parameter of sperm quality since longer motility maintenance after thawing is related to a higher pregnancy rate after AI (Bag et al., 2004).

Several years ago, the addition of HB to bull semen extender was demonstrated to reduce the membrane damage induced by osmotic stress (Hammerstedt et al., 1976), presenting a synergic protective effect with egg yolk (Graham and Hammerstedt, 1992). In sheep, a small protective function related to thermal shock was reported (Watson and Anderson, 1983), and the benefits of HB for sperm cryopreservation in sheep (Farshad et al., 2010; Palomo et al., 2017) and other species (Memon et al., 2011; Naijian et al., 2013; Merino et al., 2015) was demonstrated. Considering that cryopreservation increases the sperm sensitivity to oxidative stress (Marti et al., 2008), the effects of HB may go further than thermal shock protection, as HB is a synthetic analogue of vitamin E, an inhibitor of lipoxygenase, and able to prevent oxidation of polyunsaturated fatty acids (Bast et al., 1991; Hanada, 2014).

Although TH has an antioxidant function and prevents membrane structural damage during lipid phase transition (Najafi et al., 2013), positive effects from TH addition on both G5L2 and G5L8 extenders were not observed. Improved post-thaw sperm characteristics and fertility was reported after the addition of TH in the extender containing egg yolk (Öztürk et al., 2020; Rostami et al., 2020). However, corroborating with the present study, when LDL was used to replace egg yolk, no positive effects of TH during cryopreservation were noticed, and it was observed that it might be harmful to sperm at a high concentration (100 mM) when associated with LDL and glycerol (Tonieto et al., 2010; Varela et al., 2020).

No benefit of AA addition was detected, regardless of the LDL concentration tested, which corroborates Sönmez and Demirci (2004), who reported no protective effect of AA on thawed ram sperm and toxicity at concentration superior to 20 mM. Conversely, El-Hamid et al. (2018) reported positive effects on ram post-thaw sperm quality when AA was added to extender with 40% egg yolk, especially at low concentration (0.1 mM). Azawi and Hussein (2013) also obtained better results adding AA (5 mM) in extender with 15% egg yolk during maintenance of ram sperm at 5 °C. Due to its hydrophilic characteristic, AA presents low efficacy in preventing plasma membrane lipoperoxidation (Agarwal et al., 2016). Considering that LDL presents higher antioxidant potential than egg yolk (Vera-Munoz et al., 2011), we inferred that it might obviate identifying any benefit of AA in the present study, but no toxic effect was detected in the concentration used (10 mM).

The AP demonstrated high sperm toxicity at 10 mM. Ascorbyl palmitate is a lipophilic derivate of AA (Caritá et al., 2020) and for decades has been used as an antioxidant food additive (Zobel, 1976). After oral administration of AP, the molecule is esterified in ascorbic and palmitic acids, but the *in vitro* addition of AP inhibited cytochrome P450 activity (Dresser et al., 2002). In general, proteins from the cytochrome P450 superfamily participate in several cellular pathways (Denisov et al., 2005). The lower expression or modification in enzymatic pathways of cytochrome P450 was related to low semen quality in humans (Said et al., 2014; Yu et al., 2019), which might explain the negative effect on sperm motility after adding AP to the extender. Unfortunately, no studies with the addition of AP on media for sperm cryopreservation were found in the literature and we are not able to explain the higher percentage of thawed sperm with both plasma and acrosomal membrane integrity, but no effect on membrane functionality and impairment of sperm motility.

## Conclusion

Low-density lipoprotein at 2 and 8% was effective for replacing egg yolk in Tris-Glucose extender for ram sperm cryopreservation. Moreover, the addition of 10 mM HB in Tris-Glucose extender with 8% LDL improves velocity parameters immediately post-thaw compared with Tris-Glucose egg yolk extender, as well as prevents the reduction of total motility and VCL during incubation, whereas no benefit of adding ascorbic acid and trehalose was observed. For the first time, ascorbyl palmitate is shown to be detrimental to ram sperm motility.

## Supplementary Material

Supplementary material accompanies this paper.

Table S1 Post-thaw kinematic parameters of ram sperm (mean ± SEM) frozen in Tris-glucose base extender containing 16% egg yolk and 5% glycerol (Control) or using base extender with no egg yolk and different glycerol (G3, 5, or 7%) and low-density lipoprotein (L2, 8, or 16%) concentrations.

Table S2 Kinematic parameters of ram sperm (mean ± SEM) frozen in Tris-glucose base extender containing 16% egg yolk and 5% glycerol (Control) or using base extender with no egg yolk and different glycerol (G3, 5, or 7%) and low-density lipoprotein (L2, 8, or 16%) concentrations during 3h of incubation at 38 °C after thawing.

Figure S1 Experiment I ram sperm frozen in Tris-glucose base extender containing 16% egg yolk and 5% glycerol (Control, C) or using base extender with no egg yolk and different glycerol (G3, 5, or 7%) and low-density lipoprotein (L2, 8, or 16%) concentrations. Structural (IP^-^CFDA^+^) and functional integrity (hypoosmotic test) post-thaw. Different letters indicate differences among treatments (P < 0.05).

This material is available as part of the online article from: https://doi.org/10.1590/1984-3143-AR2022-0068
