# Supplementary material for: Post-thaw quality of ram sperm frozen with different concentrations of low-density lipoproteins associated with non-enzymatic antioxidants
Source: Anim Reprod. 2023 Apr 21;20(1):e20220068. doi: 10.1590/1984-3143-AR2022-0068 (PMC10127573; doi:10.1590/1984-3143-AR2022-0068)
Supplement: Table S1 [file 1984-3143-ar-20-1-e20220068-suppl.pdf]

10.1590/1984-3143-AR2022-0068
ORIGINAL ARTICLE
e20220068
Copyright © The Author(s). This is an Open Access article distributed under the terms of the Creative Commons Attribution License, which permits unrestricted use, distribution, and reproduction in any medium, provided the original work is properly cited.

Post-thaw quality of ram sperm frozen with different concentrations of low-density lipoproteins associated with non-enzymatic antioxidants

Paola Pereira das Neves Snoeck[1*] (http://orcid.org/0000-0003-4445-8630), Diogo Ribeiro Câmara[2] (http://orcid.org/0000-0002-9873-7419), Luís Claúdio de Oliveira Moura[1] (http://orcid.org/0000-0002-6995-9864), Maíra Corona da Silva[1] (http://orcid.org/0000-0003-3455-2789), Mariana Machado-Neves[3] (http://orcid.org/0000-0002-7416-3529), Milton Rezende Teixeira-Neto[4] (http://orcid.org/0000-0002-6516-6322), Marc Henry[5] (*In Memoriam*; http://orcid.org/0000-0003-4730-8128)

[1]Departamento de Ciências Agrárias e Ambientais da Universidade Estadual de Santa Cruz – UESC, Ilhéus, BA, Brasil

[2]Departamento de Medicina Veterinária da Universidade Federal de Alagoas - UFAL, Viçosa, AL, Brasil

[3]Departamento de Biologia Geral, Universidade Federal de Viçosa, UFV, Viçosa, MG, Brasil

[4]Departamento de Medicina Veterinária, Centro Universitário UNIFTC, Vitória da Conquista, BA, Brasil

[5]Departamento de Clínica e Cirurgia Veterinárias, Universidade Federal de Minas Gerais – UFMG, Belo Horizonte, MG, Brasil

How to cite: Snoeck PPN, Câmara DR, Moura LCO, Silva MC, Machado-Neves M, Teixeira-Neto MR, Henry M. Post-thaw quality of ram sperm frozen with different concentrations of low-density lipoproteins associated with non-enzymatic antioxidants. Anim Reprod. 2023;20(1):e20220068. DOI: https://doi.org/10.1590/1984-3143-AR2022-0068

Supplementary material

Post-thaw sperm analysis of ram semen frozen with extenders containing different concentrations of glycerol and low-density lipoproteins. Ejaculates from six rams (2-5 years) were collected (three repetitions) and individually aliquoted into ten treatments (18 samples/treatment) using a Tris-Glucose base extender: C (Control; egg yolk (16%) and glycerol (5%), 1,241 mOsmol/L); G3L2 (Tris-Glucose + glycerol (3%) + LDL (2%), 774 mOsmol/L), G5L2 (Tris-Glucose + glycerol (5%) + LDL (2%), 1,208 mOsmol/L), G7L2 (Tris-Glucose + glycerol (7%) + LDL (2%), 1,506 mOsmol/L), G3L8 (Tris-Glucose + glycerol (3%) + LDL (8%), 766 mOsmol/L), G5L8 (Tris-Glucose + glycerol (5%) + LDL (8%), 1,165 mOsmol/L), G7L8 (Tris-Glucose + glycerol (7%) + LDL (8%), 1,492 mOsmol/L), G3L16 (Tris-Glucose + glycerol (3%) + LDL (16%), 848 mOsmol/L), G5L16 (Tris-Glucose + glycerol (5%) + LDL (16%), 1,170 mOsmol/L), and G7L16 (Tris-Glucose + glycerol (7%) + LDL (16%), 1,481 mOsmol/L).

Table S1. Post-thaw kinematic parameters of ram sperm (mean ± SEM) frozen in Tris-glucose base extender containing 16% egg yolk and 5% glycerol (Control) or using base extender with no egg yolk and different glycerol (G3, 5, or 7%) and low-density lipoprotein (L2, 8, or 16%) concentrations.

Extender	Parameters						
	TM (%)	PM (%)	VCL (µm/s)	VSL (µm/s)	VAP (µm/s)	LIN (%)	ALH (µm)
Control	22.6 ± 2.0^{b}	4.1 ± 0.8^{b}	33.0 ± 1.6^{c}	12.3 ± 1.2^{b}	17.4 ± 1.3^{b}	36.2 ± 1.9^{b}	2.3 ± 0.2^{b}
G3L2	32.8 ± 2.9^{ab}	10.9 ± 1.8^{ab}	42.7 ± 1.7^{ab}	20.1 ± 1.9^{ab}	26.6 ± 2.0^{ab}	45.5 ± 2.7^{ab}	2.8 ± 0.1^{a}
G5L2	48.1 ± 5.8^{a}	15.5 ± 3.4^{a}	41.6 ± 2.1^{ab}	19.2 ± 2.0^{ab}	26.0 ± 2.3^{ab}	44.3 ± 2.3^{ab}	2.6 ± 0.1^{ab}
G7L2	23.2 ± 1.6^{b}	4.0 ± 0.4^{b}	34.4 ± 1.4^{bc}	13.4 ± 0.9^{ab}	19.2 ± 1.1^{ab}	38.4 ± 1.6^{ab}	2.5 ± 0.1^{ab}
G3L8	30.8 ± 2.3^{b}	10.3 ± 1.2^{ab}	44.2 ± 1.6^{a}	20.6 ± 1.6^{ab}	26.7 ± 1.7^{ab}	45.3 ± 2.2^{ab}	2.7 ± 0.1^{ab}
G5L8	34.2 ± 3.3^{ab}	11.1 ± 2.4^{ab}	44.1 ± 2.2^{a}	19.1 ± 1.8^{ab}	26.0 ± 1.9^{ab}	42.1 ± 1,8^{ab}	2.9 ± 0.1^{a}
G7L8	34.0 ± 4.1^{ab}	12.5 ± 3.3^{ab}	42.7 ± 2.7^{ab}	19.5 ± 2.3^{ab}	26.6 ± 2.5^{ab}	43.4 ± 2.5^{ab}	2.5 ± 0.1^{ab}
G3L16	35.5 ± 3.8^{ab}	13.9 ± 2.8^{a}	45.8 ± 2.2^{a}	22.2 ± 2.0^{a}	28.4 ± 2.0^{a}	47.4 ± 2.1^{a}	2.7 ± 0.1^{ab}
G5L16	31.3 ± 2.5^{b}	11.8 ± 2.2^{ab}	44.8 ± 2.4^{a}	21.8 ± 2.2^{a}	27.7 ± 2.3^{a}	46,9 ± 2,5^{ab}	2.7 ± 0.1^{ab}
G7L16	28.6 ± 2.3^{b}	10.4 ± 1.9^{ab}	44.4 ± 2.2^{a}	20.1 ± 1.6^{ab}	26.2 ± 1.6^{ab}	44.5 ± 1.8^{ab}	2.9 ± 0.1^{a}

TM: total motility; PM: progressive motility; VCL: curvilinear velocity; VSL: progressive velocity; VAP: path velocity; LIN: linearity; ALH: amplitude of lateral head displacement. Within columns, means with no common superscript letters are different (P < 0.05).

Table S2. Kinematic parameters of ram sperm (mean ± SEM) frozen in Tris-glucose base extender containing 16% egg yolk and 5% glycerol (Control) or using base extender with no egg yolk and different glycerol (G3, 5, or 7%) and low-density lipoprotein (L2, 8, or 16%) concentrations during 3h of incubation at 38 °C after thawing.

		**Incubation time**			
	**Extender**	**0h**	**1h**	**2h**	**3h**
**TM (%)**	Control	22.6 ± 2.0^{b}	22.2 ± 3.1^{b}	18.2 ± 2.4^{b}	15.6 ± 1.6^{a}
	G3L2	32.8 ± 2.9^{ab}	33.4 ± 2.5^{ab}	26.7 ± 3.6^{ab}	22.0 ± 2.9^{a}
	G5L2	48.1 ± 5.8^{a}	41.6 ± 5.2^{a}	33.3 ± 4.2^{a}	$25.6 \pm 3.4^{a*}$
	G7L2	23.2 ± 1.6^{b}	25.3 ± 2.0^{ab}	19.3 ± 0.9^{ab}	$18.2 \pm 1.1^{a*}$
	G3L8	30.8 ± 2.3^{b}	24.4 ± 1.9^{ab}	$20.3 \pm 0.9^{ab*}$	$17.4 \pm 1.3^{a*}$
	G5L8	34.2 ± 3.3^{ab}	29.5 ± 3.8^{ab}	29.6 ± 4.7^{ab}	19.6 ± 2.8^{a}
	G7L8	34.0 ± 4.1^{ab}	28.2 ± 3.1^{ab}	24.4 ± 4.0^{ab}	19.5 ± 1.5^{a}
	G3L16	35.5 ± 3.8^{ab}	27.4 ± 3.2^{ab}	23.0 ± 1.6^{ab}	$19.5 \pm 2.8^{a*}$
	G5L16	31.3 ± 2.5^{b}	26.8 ± 3.7^{ab}	21.6 ± 2.5^{ab}	$18.1 \pm 2.2^{a*}$
	G7L16	28.6 ± 2.3^{b}	27.0 ± 4.7^{ab}	20.4 ± 2.3^{ab}	17.6 ± 3.1^{a}
**VCL (µm/s)**	Control	33.0 ± 1.6^{c}	30.8 ± 2.2^{b}	26.8 ± 2.0^{b}	$24.1 \pm 1.6^{b*}$
	G3L2	42.7 ± 1.7^{ab}	39.1 ± 1.1^{ab}	36.9 ± 1.9^{a}	$32.5 \pm 2.6^{ab*}$
	G5L2	41.6 ± 2.1^{ab}	43.3 ± 2.3^{a}	40.2 ± 2.1^{a}	35.9 ± 2.7^{a}
	G7L2	34.4 ± 1.4^{bc}	34.8 ± 1.7^{ab}	32.6 ± 1.9^{ab}	28.9 ± 1.3^{ab}
	G3L8	44.2 ± 1.6^{a}	41.0 ± 2.6^{a}	38.4 ± 1.7^{a}	$31.0 \pm 1.5^{ab*}$
	G5L8	44.1 ± 2.2^{a}	44.3 ± 2.1^{a}	42.0 ± 2.8^{a}	38.0 ± 4.0^{a}
	G7L8	42.7 ± 2.7^{ab}	43.4 ± 3.9^{a}	43.1 ± 4.1^{a}	35.2 ± 2.7^{a}
	G3L16	45.8 ± 2.2^{a}	43.4 ± 3.3^{a}	40.1 ± 2.3^{a}	35.0 ± 3.5^{ab}
	G5L16	44.8 ± 2.4^{a}	44.5 ± 3.5^{a}	35.0 ± 2.5^{ab}	39.4 ± 3.5^{a}
	G7L16	44.4 ± 2.2^{a}	44.2 ± 3.4^{a}	40.1 ± 4.4^{a}	37.8 ± 4.3^{a}
**LIN (%)**	Control	36.2 ± 1.9^{b}	28.9 ± 3.0^{b}	24.6 ± 2.5^{b}	26.9 ± 3.5^{a}
	G3L2	45.5 ± 2.7^{ab}	39.6 ± 2.2^{ab}	37.0 ± 2.6^{a}	33.5 ± 3.5^{a}
	G5L2	44.3 ± 2.3^{ab}	42.2 ± 2.5^{a}	40.5 ± 2.0^{a}	38.4 ± 3.2^{a}
	G7L2	38.4 ± 1.6^{ab}	37.6 ± 2.8^{ab}	37.8 ± 2.8^{a}	36.4 ± 2.0^{a}
	G3L8	45.3 ± 2.2^{ab}	37.6 ± 2.9^{ab}	34.3 ± 2.7^{ab}	$29.0 \pm 2.5^{a*}$
	G5L8	42.1 ± 1.8^{ab}	35.4 ± 1.5^{ab}	37.3 ± 2.2^{a}	$32.5 \pm 2.5^{a*}$
	G7L8	43.4 ± 2.5^{ab}	33.1 ± 2.3^{ab}	31.1 ± 2.4^{ab}	$31.5 \pm 2.8^{a*}$
	G3L16	47.4 ± 2.1^{b}	34.1 ± 2.3^{ab}	34.2 ± 1.5^{ab}	$33.7 \pm 2.5^{a*}$
	G5L16	46.9 ± 2.5^{ab}	34.0 ± 1.8^{ab}	33.3 ± 2.1^{ab}	$32.5 \pm 1.4^{a*}$
	G7L16	44.5 ± 1.8^{ab}	32.0 ± 1.7^{ab}	30.4 ± 1.8^{ab}	$31.0 \pm 2.0^{a*}$

Within columns, means with no common superscript letters are different (P < 0.05). * Indicates reduction within treatment during incubation time (P < 0.05).

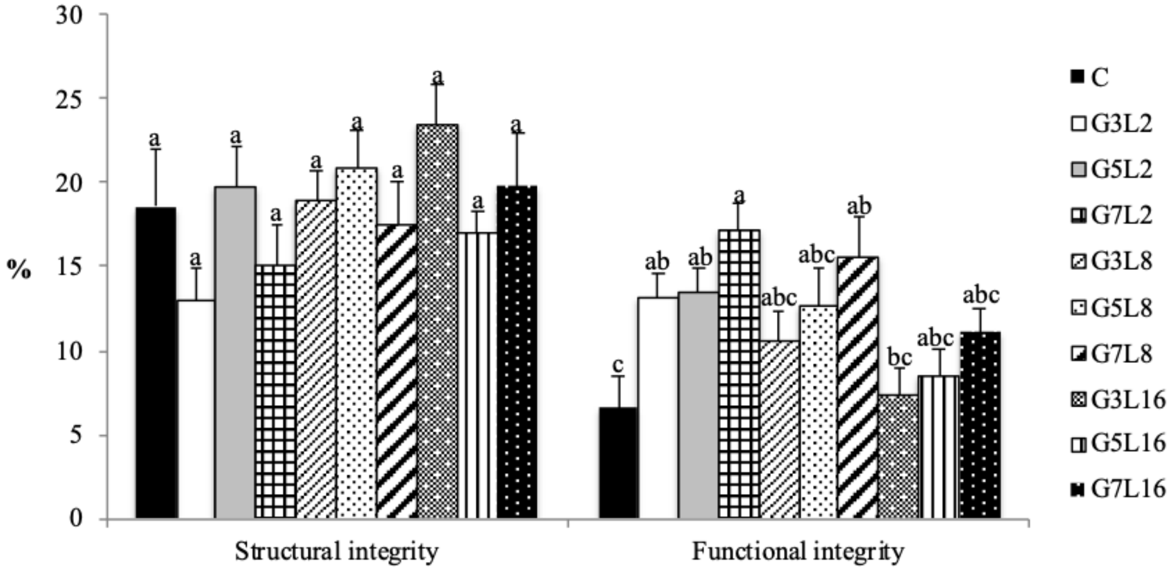

Figure S1. Experiment I ram sperm frozen in Tris-glucose base extender containing 16% egg yolk and 5% glycerol (Control, C) or using base extender with no egg yolk and different glycerol (G3, 5, or 7%) and low-density lipoprotein (L2, 8, or 16%) concentrations. Structural ($IP^{-}CFDA^{+}$) and functional integrity (hypoosmotic test) post-thaw. Different letters indicate differences among treatments ($P < 0.05$).
